# Mineral Bone Disease Prevalence and Biochemical Profile in Chronic Kidney Disease Patients Undergoing Hemodialysis

**DOI:** 10.7759/cureus.84747

**Published:** 2025-05-24

**Authors:** Jaison George, Rammohan S Bhat

**Affiliations:** 1 Department of Nephrology, Malankara Orthodox Syrian Church Medical College, Kochi, IND; 2 Department of Nephrology, Kauvery Hospitals, Bengaluru, IND

**Keywords:** alkaline phosphatase (alp), bone-specific alkaline phosphatase, intact parathyroid hormone, maintenance hemodialysis, mineral bone disease

## Abstract

Introduction

Mineral bone disease (MBD) is a common complication in patients with stage 5 chronic kidney disease (CKD) undergoing maintenance hemodialysis. This study aimed to evaluate the prevalence and biochemical profiles of MBD in this patient population in India.

Methods

This cross-sectional study was conducted at a tertiary hospital in southern India over a period from June 2020 to November 2021 with 152 patients with stage 5 chronic kidney disease (CKD) undergoing maintenance hemodialysis for at least three months. Blood samples were collected prior to routine dialysis sessions to assess levels of calcium, phosphate, intact parathyroid hormone (iPTH), 25-hydroxy vitamin D, bone-specific alkaline phosphatase (BSAP), and total alkaline phosphatase (ALP). Patients were categorized based on their iPTH levels into four distinct groups: < 2 times, 2 to 5 times, 5 to 9 times, and > 9 times the upper normal limit (which is 65 pg/ml).

Results

Low-turnover MBD (iPTH < 2 times the upper limit) was predominant with 66 (43.4%) patients, whereas 17 (11.18%) patients had high-turnover MBD (iPTH > 9 times the upper limit). Hypocalcemia and hypercalcemia affected 42 (27.6%) and 14 (9.2%) patients, respectively. Vitamin D insufficiency was found in 88(57.9%) patients, and deficiency was found in 18 (11.8%) patients. BSAP levels were predominantly high (≥27 IU/L) across all iPTH groups, with no statistically significant variation. In contrast, total alkaline phosphatase (ALP) levels showed a significant positive correlation with iPTH levels (p=0.001), with higher iPTH levels associated with a greater proportion of ALP values > 110 IU/L. These findings underscore the high prevalence of MBD in Indian patients with CKD on hemodialysis, emphasizing the need for biochemical monitoring to facilitate early detection and optimal management for improved outcomes.

Conclusion

The high prevalence of MBD in Indian patients with CKD on hemodialysis underscores the need for regular biochemical monitoring. Early detection and optimal management strategies are essential to improve patient outcomes.

## Introduction

In India, approximately 17% of adults have chronic kidney disease (CKD), a significant public health concern [1]. CKD prevalence varies across India and is affected by urbanization, socioeconomic conditions, and access to healthcare [2]. Mineral bone disease (MBD) complicates 60-100% of CKD cases, with a higher incidence in stages 4 and 5. Skeletal complications are nearly universal in patients with CKD undergoing dialysis (stage 5D), and are common in stages 3-5 [3]. In patients with CKD, MBD involves disturbances in bone metabolism, often causing bone pain, fractures, and cardiovascular diseases. The etiology of MBD in India is complex and includes inadequate nutrition, vitamin D deficiency, high phosphate intake from traditional diets, and limited access to phosphate binders and vitamin D supplements. These issues, coupled with delayed CKD diagnosis, contribute to the high prevalence and severity of MBD in this population [4].

Although treatments for secondary and tertiary hyperparathyroidism, including phosphate binders, vitamin D analogues, and calcimimetics, are available, the management of MBD in CKD patients remains suboptimal. The challenges in managing MBD in India are exacerbated by diagnostic difficulties, including the limited availability of specialized tests and high costs of monitoring calcium, phosphate, and parathyroid hormone levels. Understanding the prevalence and impact of MBD in patients with CKD on dialysis is essential for developing effective management strategies and improving outcomes.

This study aimed to examine the prevalence of MBD in patients with CKD undergoing dialysis, focusing on unique challenges and risk factors within the Indian healthcare context. By elucidating the extent of this issue, this study seeks to contribute to developing tailored guidelines, raising awareness among healthcare providers and patients, and encouraging research on cost-effective interventions for resource-limited settings. Intact parathyroid hormone (iPTH) is crucial for identifying mineral bone diseases (MBD) because bone biopsy is invasive. iPTH levels provide insights into bone metabolism, distinguishing high-turnover bone diseases (e.g., secondary hyperparathyroidism) from low-turnover bone diseases (e.g., adynamic bone disease) [5]. Elevated iPTH levels typically suggest increased bone resorption, whereas low iPTH levels indicate reduced bone activity. However, its diagnostic accuracy is limited [6].

Research has shown that the inclusion of iPTH in combination with biochemical markers such as bone-specific alkaline phosphatase (BSAP) and alkaline phosphatase (ALP) improves MBD classification and facilitates patient-specific treatment [7]. This study aimed to assess the biochemical profile of such patients to determine the prevalence and investigate the role of other biochemical markers, in combination with iPTH, in enhancing the classification of MBD.

## Materials and methods

Study design and population

This cross-sectional study was conducted at Mazumdar Shaw Medical Center, Narayana Health City, a tertiary care center, located in Bengaluru, India, from June 2020 to November 2021. A total of 152 patients undergoing maintenance hemodialysis for at least three months were included in the study.

Sample Size Calculation

Based on the expected prevalence of mineral bone disorder (85%) from a study by Ahmed et al, with a 95% confidence level and 5% margin of error, the minimum required sample size was calculated as 196 patients. While we could only include 152 patients due to the COVID-19 pandemic, this sample size still achieves a margin of error of 5.7%, which is close to our target precision. The calculation used the formula:

$n = \frac{z^2 p (1 - p)}{E^2}$

Where z = 1.96 (95% confidence level), p = 0.85 (expected prevalence), and E = 0.05 (margin of error).

Using a two-sided test comparing our observed prevalence (85%) against a baseline prevalence of 80%, the effect size (Cohen's h) was 0.132. The statistical power achieved was 21%. While our achieved sample size was lower than initially planned due to pandemic-related constraints, it still allowed us to estimate the prevalence with reasonable precision (5.7% margin of error). However, we acknowledge this as a limitation of our study, particularly regarding the statistical power to detect smaller differences in prevalence.

Inclusion Criteria

The study included adults with chronic kidney disease stage 5, aged 18 years or older, who were receiving maintenance hemodialysis. Eligible participants were those who had been on stable hemodialysis for at least three months and were willing to provide written informed consent.

Exclusion Criteria

Individuals were excluded if they had acute kidney injury requiring temporary dialysis, known malignancies, or active infections. Patients receiving medications that affect bone metabolism, such as bisphosphonates or corticosteroids, were also excluded. However, those on regular phosphate binders, calcium, and calcium analogues were not disqualified from participation.

Sampling technique

A simple random sampling method was employed to select participants from the total patient pool undergoing maintenance hemodialysis at the hospital during the study period. A complete list of eligible patients was obtained from hospital records, and participants were randomly selected using a lottery system to ensure unbiased selection.

Biochemical measurements

Blood samples were collected prior to regular dialysis session to assess levels of calcium (reference normal range of 8.5-10.5 mg/dL), phosphate (normal reference range of 2.5-4.5 mg/dL), intact parathyroid hormone (iPTH, normal reference range of 10-65 pg/mL), 25-hydroxy vitamin D (normal reference range ≥30ng/ml), bone-specific alkaline phosphatase (BSAP), and total alkaline phosphatase (ALP) at once and no repeat sampling were done. The upper limit of normal for iPTH was set at 65 pg/ml, with upper normal levels for BSAP and ALP defined as 27 IU/L and 110 IU/L, respectively. Vitamin D levels between 20-30 ng/mL were classified as insufficient, while levels below 20 ng/mL were considered deficient.

Patients were categorized based on their iPTH levels into four distinct groups. The low turnover group consisted of individuals with iPTH levels below twice the upper normal limit, specifically less than 130 pg/ml. The second group included those with iPTH levels ranging from 2 to 5 times the upper normal limit, falling within the range of 130 to 325 pg/ml. Patients with iPTH levels 5 to 9 times the upper normal limit, ranging from 325 to 585 pg/ml, belonged to the third group. Finally, the high turnover group encompassed individuals with iPTH levels > 9 times the upper normal limit, which translated to values above 585 pg/ml.

Statistical analysis

Data analysis was carried out using SPSS software version 20 (IBM Corp., Armonk, USA). We assessed the normality of continuous variables using the Shapiro-Wilk test. For normally distributed variables, we used parametric tests and presented results as mean ± standard deviation, while for non-normally distributed variables, we used non-parametric tests and presented results as median with interquartile range. The chi-square test was applied to categorical variables, while Analysis of Variance (ANOVA) was used for continuous data. Statistical significance was considered at p < 0.05.

Ethical considerations

The study was reviewed and approved by the Academic Ethics Committee of Narayana Health prior to initiation (No. NHH/AEC-CL-2020-552). Written informed consent was obtained from all participants, ensuring compliance with ethical principles outlined in the Declaration of Helsinki.

## Results

The mean age of participants was 51.91 ± 14.10 years. Hemodialysis vintage varied widely, with a mean duration of 36.05 ± 27.21 months and a median of 30 months (Table 1). 

**Table 1:** Continuous baseline characteristics

Variables			Values (n)
Age (mean ± SD)			51.91±14.10 years
Hemodialysis vintage (months)		(mean ± SD)	36.05±27.21
		Median [Q3-Q1]	30.0 [17.2 – 46.0]

Gender distribution showed a predominance of males (78.3%) compared to females (21.7%). The majority of patients (80.9%) underwent hemodialysis thrice weekly, while 19.1% received dialysis twice weekly. A small proportion of patients had experienced fractures (1.3%) or were on medications such as vitamin D analogues (6.6%), calcium supplements (5.3%), phosphate binders (3.9%), and anticoagulants (0.7%) (Table 2).

**Table 2:** Categorical baseline characteristics

Variables			Values (n)	Percentage (%)
Gender distribution	Males		119	78.3%
	Females		33	21.7%
Frequency of hemodialysis	Thrice weekly		123	80.9%
	Twice weekly		29	19.1%
Fractures			2	1.3%
Patients on vit D analogue			10	6.6%
Patients on calcium supplements			8	5.3%
Patients on phosphate binders			6	3.9%
Patients on anticoagulants			1	0.7%

The mean serum calcium level was 9.07 ± 1.02 mg/dL, while the mean serum phosphorus level was 3.65 ± 1.52 mg/dL. Serum vitamin D levels exhibited considerable variability, with a mean of 26.39 ± 14.65 mg/dL. Parathyroid hormone (iPTH) levels showed a broad distribution, with a median of 189.30 pg/mL, reflecting variations in secondary hyperparathyroidism among patients. Bone-specific alkaline phosphatase (BSAP) had a median value of 73.08 IU/L, while total alkaline phosphatase (ALP) levels had a median of 112.50 IU/L. Median serum vitamin D levels were 23.12 ng/mL, highlighting the prevalence of vitamin D deficiency among the cohort (Table 3).  

**Table 3:** Lab parameters IPTH-Intact parathyroid hormone, BSAP-Bone specific alkaline phosphatase, ALP-Total alkaline phosphatase

Lab parameters	Values
Serum calcium (mean±SD)	9.07±1.02 mg/dl
Serum phosphorus (mean±SD)	3.65±1.52 mg/dl
Serum vitamin D (mean±SD)	26.39±14.65 mg/dl
IPTH Median [Q3-Q1]	189.30 [76.0 – 337.0] pg/ml
BSAP Median [Q3-Q1]	73.08 [66.6 – 78.7] IU/L
ALP Median [Q3-Q1]	112.50 [82.5 – 158.7] IU/L
Vitamin D Median [Q3-Q1]	23.12 [14.7 – 33.6] ng/ml

Among the study population, 66 (43.4%) patients were classified into the low-turnover group, characterized by iPTH levels less than twice the normal value, and 17 (11.18%) patients were identified as belonging to the high-turnover group, exhibiting iPTH levels > 9 times the upper limit of normal. Additionally, 46 (30.2%) patients had iPTH levels ranging from 2 to 5 times the upper limit of normal, whereas 23 (15.1%) patients had levels ranging from 5 to 9 times the upper limit of normal. 96 (63.2 %) patients from the study population had normal calcium levels, while 42 (27.6%) had hypocalcemia, and 14 (9.2%) had hypercalcemia. Comparison of calcium levels using one-way ANOVA showed that the mean value of calcium was highest in the low turnover group with iPTH < 2 times (mean value of 9.43), followed by the high turnover group with iPTH > 9 times (mean value of 8.85), iPTH 5 to 9 times (mean value of 8.84), and iPTH 2 to 5 times (mean value of 8.76). This difference was statistically significant (p-value = 0.001) (Table 4).

**Table 4:** Comparison of calcium levels with iPTH levels (one-way ANOVA test) iPTH-Intact parathyroid hormone

iPTH LEVEL	N	MEAN	STD. DEVIATION	STATISTICS/MEAN SQUARES	DF2 (WELCH)/F (ANOVA)	P-VALUE
< 2 times	66	9.427273	0.932085	6.2	47.796	0.001
2-5 times	46	8.763043	0.726746			
5-9 times	23	8.843478	1.42281			
> 9 times	17	8.847059	1.021713			
Total	152	9.073026	1.017266			

Post hoc analysis revealed that the significant overall difference in calcium levels among the groups was primarily driven by the difference between the iPTH < 2 times group (the low turnover group) and the iPTH 2 to 5 times group. Comparisons with other groups were not significant (Table 5).

**Table 5:** Post hoc analysis of calcium levels across iPTH groups (Tukey's HSD Test) iPTH: Intact parathyroid hormone, HSD: honestly significant difference

COMPARISON	MEAN DIFFERENCE	95% CI LOWER	95% CI UPPER	P-VALUE	SIGNIFICANT
iPTH 2-5 times vs iPTH 5-9 times	0.188	-0.445	0.820	0.867	No
iPTH 2-5 times vs iPTH < 2 times	0.565	0.090	1.041	0.013	Yes
iPTH 2-5 times vs iPTH > 9 times	0.130	-0.573	0.832	0.963	No
iPTH 5-9 times vs iPTH < 2 times	0.378	-0.222	0.977	0.361	No
iPTH 5-9 times vs iPTH > 9 times -	0.058	-0.850	0.734	0.998	No
iPTH < 2 times vs iPTH > 9 times -	0.435	-1.109	0.238	0.338	No

**Figure 1:**
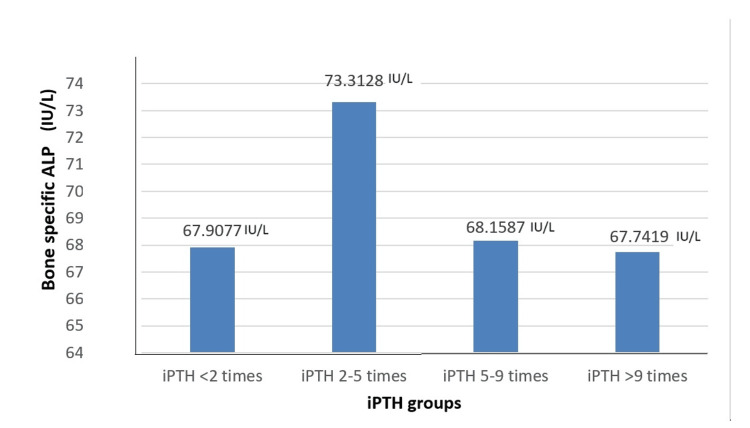
Mean values of bone specific alkaline phosphatase levels in the various iPTH groups iPTH-Intact parathyroid hormone, ALP-alkaline phosphatase

In the study population, 76 (50%) patients had normal phosphate levels, 38 (25%) had hypophosphatemia, and 38 (25%) had hyperphosphatemia. 145 (96%) had bone-specific alkaline phosphatase (BSAP) levels ≥ 27 IU/L, while six (4%) had values < 27 IU/L. A one-way ANOVA test comparing BSAP levels revealed that the mean BSAP value was the highest in the group with iPTH levels 2 to 5 times the upper limit of normal (73.31 IU/L), followed by the group with iPTH levels 5 to 9 times (68.16 IU/L), the group with iPTH levels < 2 times (67.90 IU/L), and the group with iPTH levels > 9 times (67.74 IU/L) (Figure 1).

**Figure 2:**
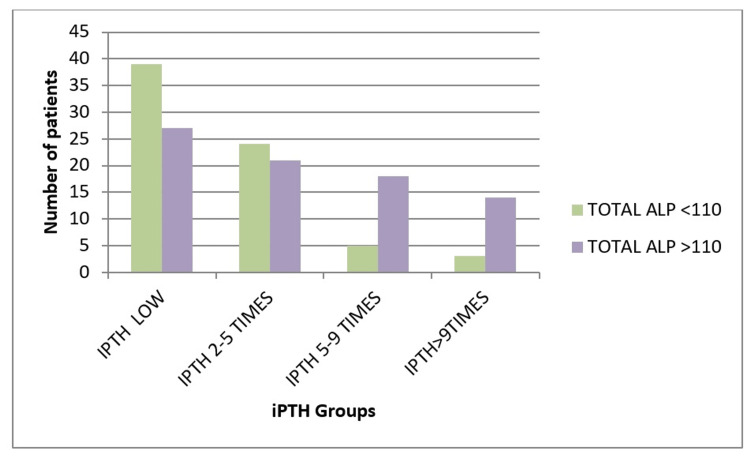
Alkaline phosphatase level with iPTH levels ALP-Alkaline phosphatase, IPTH-Intact parathyroid hormone

In this study, 71 (46.7%) of the participants had alkaline phosphatase (ALP) values ≤ 110 IU/L, while 81 (53.3%) had levels > 110 IU/L. The differences in ALP levels between the various groups were statistically significant (p = 0.001). A comparison of ALP with iPTH levels using a one-way ANOVA test indicated that the mean ALP value was highest in the > 9 times normal iPTH group (283.53 IU/L), followed by the group with iPTH levels 5 to 9 times the upper limit of normal (155.09 IU/L), the group with iPTH levels 2 to 5 times (138.22 IU/L), and the < 2 times iPTH group (121.92 IU/L). This difference was statistically significant (p=0.023) (Figure 2, Table 6).

**Table 6:** Comparison of alkaline phosphatase levels with iPTH levels (one-way ANOVA test) iPTH: Intact parathyroid hormone

iPTH LEVEL	N	MEAN	STD. DEVIATION	STATISTICS/MEAN SQUARES	DF2 (WELCH)/F (ANOVA)	P-VALUE
iPTH <2 times	66	121.92	73.759	3.48	46.943	0.023
iPTH 2-5 times	46	138.22	110.971			
iPTH 5-9 times	23	155.09	73.69			
iPTH >9 times	17	283.53	232.729			
Total	152	149.95	122.23			

Post hoc analysis using Tukey's Honestly Significant Difference (HSD) test revealed significant differences in ALP levels between iPTH groups. The iPTH > 9 times group showed significantly higher ALP levels compared to all other groups: versus iPTH < 2 times group (mean difference = 167.86 IU/L, 95% CI: 137.19 to 198.52, p < 0.001), versus iPTH 2 to 5 times group (mean difference = 148.14 IU/L, 95% CI: 116.14 to 180.13, p < 0.001), and versus iPTH 5 to 9 times group (mean difference = 126.33 IU/L, 95% CI: 90.27 to 162.39, p < 0.001). Additionally, the iPTH 5 to 9 times group demonstrated significantly higher ALP levels compared to the iPTH < 2 times group (mean difference = -41.52 IU/L, 95% CI: -68.82 to -14.23, p < 0.001). The differences between the iPTH 2 to 5 times and iPTH 5 to 9 times groups (p = 0.205), and between the iPTH 2 to 5 times and iPTH < 2 times groups (p = 0.088) were not statistically significant (Table 7).

**Table 7:** Post hoc analysis of ALP levels across iPTH groups (Tukey's HSD Test) CI-Confidence interval, ALP-Alkaline phosphatase, iPTH-Intact parathyroid hormone

COMPARISON	MEAN DIFFERENCE	95% CI INTERVAL (LOWER, UPPER)	P-VALUE
iPTH 2-5 times vs iPTH 5-9 times	21.8	(-6.99, 50.59)	0.205
iPTH 2-5 times vs iPTH < 2 times	-19.72	(-41.37, 1.93)	0.088
iPTH 2-5 times vs iPTH > 9 times	148.14	(116.14, 180.13)	< 0.001
iPTH 5-9 times vs iPTH < 2 times	-41.52	(-68.82, -14.23)	< 0.001
iPTH 5-9 times vs iPTH > 9 times -	126.33	(90.27, 162.39)	< 0.001
iPTH < 2 times vs iPTH > 9 times -	167.86	(137.19, 198.52)	< 0.001

BSAP levels were predominantly high (≥ 27 IU/L) across all iPTH groups, with no statistically significant variation. In contrast, ALP levels showed a significant positive correlation with iPTH levels (p = 0.001), with higher iPTH levels associated with a greater proportion of ALP values > 110 IU/L. Within the study population, only 46 (30.3%) had normal vitamin D levels, 88 (57.9%) exhibited insufficiency, and 18 (11.8%) had deficiency.

Within the study population, only 46 (30.3%) had normal vitamin D levels, 88 (57.9%) exhibited insufficiency, and 18 (11.8%) had deficiency.

We also investigated comorbid conditions associated with MBD. Within the study cohort, 57 (37.5%) had a history of diabetes. The highest prevalence of diabetes was observed in the high-turnover group, comprising nine (52.9%) patients with intact parathyroid hormone (iPTH) levels > 9 times the upper limit of normal. Among those with iPTH levels less than twice the upper limit, 25 (37.9%) had diabetes, followed by 17 (37%) in the group with iPTH levels 2 to 5 times, and six (26.1%) in the group with iPTH levels 5 to 9 times the upper limit. Out of the 152 patients, 15 (9.9%) had a history of coronary artery disease. Patients with a history of coronary artery disease was the highest in patients with iPTH levels 5 to 9 times the upper limit of normal, accounting for three (13%) patients of this subgroup, followed by eight (12.1%) in the group with iPTH levels less than twice the upper limit, and four (8.7%) in the group with iPTH levels 2 to 5 times the upper limit of normal of iPTH. Additionally, two patients had a history of fractures; one belonged to the high turnover group with iPTH > 9 times the normal upper limit, while the other had iPTH levels in the range of 5-9 times the upper limit of normal.

## Discussion

Mineral bone disease is a significant concern in chronic kidney disease (CKD) patients undergoing hemodialysis, and its classification plays a crucial role in guiding treatment strategies. This study aimed to evaluate the utility of biochemical markers - intact parathyroid hormone (iPTH), bone-specific alkaline phosphatase (BSAP), and alkaline phosphatase (ALP) - in distinguishing bone turnover states.

The mean age of our study population was 51.91 years, not much different from other similar Indian studies done in the past [8]. Males outnumbered females in our study - 119 (78%) vs. 33 (22%) - which aligns with trends observed in India [9]. This might reflect a systematic bias in access and utilization of healthcare facilities by females in India, likely due to socio-cultural reasons or due to a higher incidence of lifestyle diseases in males. While a few Western studies often report near-equal gender representation, international data suggest that male patients are more prevalent among those with end-stage renal disease (ESRD) in many countries [10]. This pattern is documented in global ESRD reports, including the United States Renal Data System (USRDS) Annual Data Report [11].

Various studies have shown that bone histomorphometry correlates well with iPTH values. One such study was conducted by Lena van Doorn et al. in 1993, in which transiliac bone biopsies were obtained from 24 patients (nine men and 15 women) with primary hyperparathyroidism but normal renal function [12]. They found that cortical porosity and the parameters of bone turnover correlate well with serum PTH concentration. Another study by Sprague et al. in 492 dialysis patients from Brazil, Portugal, Turkey, and Venezuela compared iPTH with prior bone biopsy and showed that iPTH could differentiate between high and low turnover states. In the same study, 50% were found to have low bone turnover on bone biopsy, while 17% had high turnover [13].

BSAP is a less commonly used biochemical parameter. Sprague et al suggested a cutoff of < 33.1 IU/L in predicting low bone turnover disease [13]. A study published by Bervoets et al in 2003 showed a good sensitivity (72%) and specificity (76%) of low bone-specific alkaline phosphatase (values less than 25) in identifying adynamic bone disease on comparison with bone histology [14]. Another study by M.M. Couttenye et al on 103 dialysis patients comparing bone biopsy findings with BSAP results showed that the combination of low PTH levels and low BSAP levels (< 27 IU/L) improved the specificity of diagnosing adynamic bone disease [15]. Hence, in our study, we used a cutoff of 27 IU/L. These studies form the basis for our study, where we have set out to use the combination of iPTH and BSAP to differentiate low- versus high-turnover bone disease. In our study, BSAP levels are predominantly high across all iPTH groups; iPTH variations do not appear to meaningfully or significantly affect BSAP levels in our study population. This suggests that other factors might be influencing BSAP levels, or that the relationship between these two markers is complex and not linear. 

Hypocalcemia and hyperphosphatemia are recognized biochemical disturbances in end-stage renal disease (ESRD), linked to secondary hyperparathyroidism and complications. Foley et al. (1996) described these abnormalities as characteristic of ESRD, emphasizing their impact on patient morbidity and mortality. Their study highlighted the association between chronic hypocalcemia and increased mortality and morbidity, underscoring the importance of maintaining calcium homeostasis [16].

In our study, 42 of 152 patients (27.6%) exhibited hypocalcemia, showing its substantial prevalence in ESRD. This finding aligns with research highlighting altered calcium metabolism due to impaired renal function and disrupted vitamin D activation. The incidence of hyperphosphatemia in our cohort was 38 patients (25%), which appears lower than rates reported in some studies. This variation may be due to dietary phosphorus intake, dialysis adequacy, phosphate binder usage, or differences in patient management strategies.

Abnormalities in vitamin D metabolism play a major role in the pathogenesis of secondary hyperparathyroidism in chronic kidney disease. Chronic kidney disease is associated with a high incidence of nutritional vitamin D insufficiency or deficiency as manifested by decreased levels of 25-hydroxyvitamin D [17]. In the study done by Z. Jabbar et al on CKD stage 4 and 5 patients and published in 2013, 80% of patients had vitamin D deficiency and 13% had insufficiency [18]. In another study conducted by the same author, consisting of 100 stage 4 and stage 5 CKD patients and 72 controls, only 1% of CKD patients and 4% of controls had normal vitamin D levels. Hence, this study showed that vitamin D deficiency was widely prevalent in the general population and was even worse in those with CKD [19]. In our study, too, only 14 patients out of 152 (9.2%) had normal levels of vitamin D. This can be explained by the fact that, in addition to CKD, the vitamin D content of the traditional Indian diet, which is mostly vegan in nature, is low. This could also be due to changing lifestyle patterns that have reduced the amount of sun exposure, as most people are now engaged in indoor activities [20].

The majority of patients in our study population were presumed to have chronic glomerulonephritis, followed by diabetic nephropathy. Renal biopsies were not performed for the vast majority of these patients, as most people at the time of diagnosis were unsuitable for biopsy. Globally, diabetic nephropathy is the most common cause of CKD [21, 22].

## Conclusions

This study demonstrates a significant prevalence of mineral bone disease among patients with stage 5 chronic kidney disease undergoing maintenance hemodialysis in India, highlighting the need for regular monitoring of biochemical parameters.

The integration of iPTH, BSAP, and ALP as biochemical tools presents a non-invasive approach for assessing bone turnover states in CKD patients undergoing dialysis. While bone biopsy remains the gold standard, these markers provide valuable clinical insights that can inform therapeutic strategies. Future research with longitudinal follow-up and larger sample sizes will be essential to further validate these biochemical interactions and improve classification models for mineral bone disease in CKD.
